# Residual Periodontal Pockets at Implant Placement as Risk Indicator for Peri‐Implantitis: A Systematic Review

**DOI:** 10.1111/cid.70174

**Published:** 2026-07-27

**Authors:** Francesco Ferrarotti, Giacomo Baima, Alessandro Campagna, Matteo Corana, Enrico Cafasso, Sompol Chuachamsai, Melanie Llerena‐Velasquez, Giulia Maria Mariani, Mario Aimetti

**Affiliations:** ^1^ Department of Surgical Sciences, C.I.R. Dental School University of Turin Turin Italy; ^2^ Department of Biomedical, Metabolic and Neural Sciences University of Modena and Reggio Emilia Modena Italy

**Keywords:** dental implants, furcation involvement, peri‐implantitis, periodontitis, residual periodontal pockets, risk factors, supportive periodontal therapy

## Abstract

**Intro:**

The present systematic review aimed to assess whether the presence of residual periodontal pockets (PPD) after active periodontal therapy increases the risk of developing peri‐implantitis in patients with treated periodontitis who undergo implant therapy.

**Methods:**

An electronic search was performed in MEDLINE via PubMed, Scopus, Embase, and Cochrane Library. Studies including patients receiving at least one dental implant, in whom residual PPD or furcation involvement were recorded, were selected. The primary outcome was the incidence of peri‐implantitis. Risk of bias was assessed using the Newcastle–Ottawa Scale. No meta‐analysis could be performed due to heterogeneity across studies.

**Results:**

From 3870 retrieved records, 4 cohort studies fulfilled the inclusion criteria, with follow‐up ranging from 1 to 23 years. All included studies evaluated residual PPD as the exposure of interest, whereas no study specifically assessed furcation involvement. Despite substantial heterogeneity in exposure definitions and peri‐implantitis case definitions, all studies consistently suggested that residual PPD persisting after active periodontal therapy was associated with increased peri‐implantitis occurrence. In particular, deeper PPD (≥ 5 or ≥ 6 mm) and higher proportions of residual diseased sites were associated with greater peri‐implantitis risk. One study additionally reported that inflammatory periodontal conditions affecting adjacent teeth increased peri‐implantitis risk. Most studies were judged to be at moderate risk of bias.

**Conclusion:**

Residual PPD persisting after active periodontal therapy can increase the risk of peri‐implantitis in treated periodontitis patients receiving dental implants. Prospective studies adopting standardized case definitions of peri‐implantitis are needed to further clarify the influence of residual PPDs and furcation involvement on implant outcomes.

**Trial Registration:** PROSPERO: CRD42024567630

## 1 Introduction

Peri‐implantitis is a primary biological complication of implant therapy, characterized by an inflammatory reaction in the peri‐implant mucosa accompanied by progressive loss of supporting bone [1, 2]. Epidemiological studies indicate that more than one‐third of patients and one‐fifth of implants are affected by peri‐implantitis [3, 4, 5], underscoring the importance of primordial and primary prevention strategies [6]. Consequently, the identification and control of risk factors remain central to contemporary implant therapy [6, 7].

Among the factors most consistently associated with peri‐implantitis are a history of periodontitis, inadequate plaque control, tobacco smoking, uncontrolled diabetes, implant malpositioning, and irregular adherence to supportive periodontal/peri‐implant care (SPC/SPIC) [4, 5, 8]. Within this framework, periodontitis emerges as one of the most robust and consistently validated risk indicators [6, 9, 10, 11]. At the same time, because periodontitis remains the leading cause of tooth loss in adults [12, 13], a substantial proportion of implant therapy is inevitably performed in patients previously affected by the disease [14, 15]. Current clinical guidelines recommend achieving periodontal stability before implant placement [7, 16]. However, in routine clinical practice, the complete resolution of deep periodontal pockets and furcation involvement is frequently difficult to accomplish, particularly in patients with advanced disease and complex anatomical defects [17, 18].

To date, most primary studies and systematic reviews have evaluated periodontitis mainly in terms of patient history or periodontal status assessed at the time of peri‐implantitis diagnosis [3, 4, 14, 19, 20]. Comparatively, less attention has been directed toward the persistence of residual PPDs, furcation‐involved teeth, or incomplete achievement of periodontal treatment endpoints at the time of implant placement [21]. Such conditions may reflect residual inflammatory burden and ecological dysbiosis following periodontal therapy, while simultaneously serving as microbial reservoirs capable of contributing to early peri‐implant colonization and inflammation [22, 23].

Against this background, the present systematic review aims to address the following focused question: “In patients with treated periodontitis receiving at least one dental implant, do residual PPDs (> 4 mm) and/or furcation‐involved teeth (FI) ≥II degree at the time of implant placement increase the risk of peri‐implantitis compared with patients exhibiting periodontal stability or periodontal health?”

## 2 Materials and Methods

This systematic review was conducted in accordance with the PRISMA (Preferred Reporting Items for Systematic Reviews and Meta‐analyses) guidelines and checklist [24, 25] and was prospectively registered in the PROSPERO database (CRD42024567630).

### 2.1 Inclusion Criteria

- Population (P): Human patients rehabilitated with at least one dental implant, and for whom a full‐mouth periodontal examination was performed and reported after active periodontal therapy and prior to implant placement/loading.
- Exposure (E): Implant placement in patients presenting residual PPD > 4 mm after active periodontal therapy and/or FI ≥ II.
- Comparison (C): Implant placement in patients without residual PPDs or FI ≥ II after active periodontal therapy.
- Outcomes (O):
  ○ Primary outcome: Incidence of peri‐implantitis.
  ○ Secondary outcomes: Implant loss; incidence of mucositis; changes in peri‐implant clinical parameters (PPD, plaque index, bleeding index); and marginal bone level (MBL) changes.
- Study designs (S): Randomized controlled clinical trials (RCTs), controlled clinical trials (CCTs), prospective cohort studies, retrospective studies, and case series with a minimum of 1‐year follow‐up after implant loading.

### 2.2 Exclusion Criteria

- Studies recruit exclusively patients with diabetes or other conditions affecting bone metabolism.
- Studies recruiting exclusively smoking individuals.
- Single case reports, animal experiments, and ex vivo or in vitro studies.

### 2.3 Search Strategy

An electronic search was performed across four databases: MEDLINE (via PubMed), Scopus, Cochrane CENTRAL, and Embase up to December 2025. Both MeSH terms and free‐text keywords were combined using Boolean operators (AND, OR). The complete search strategies for each database are reported in Supporting Information 1.

Additionally, a manual search was conducted in the following journals for the last 5 years: *Journal of Periodontology*, *Journal of Clinical Periodontology*, *Journal of Dental Research*, *Journal of Periodontal Research*, *Clinical Oral Implants Research*, *International Journal of Periodontics and Restorative Dentistry*, *International Journal of Oral and Maxillofacial Implants*, and *Clinical Implant Dentistry and Related Research*. Reference lists of included studies and relevant reviews were also screened.

### 2.4 Screening and Study Inclusion

Two reviewers (A.C. and M.C.) independently screened the titles and abstracts in duplicate after calibration on a random sample of 50 citations. Discrepancies were resolved by consensus or by consultation with a third reviewer (G.B.). The same procedure was applied for full‐text screening.

All references were managed in Rayyan [26], and duplicates were removed automatically and manually. Inclusion/exclusion decisions were recorded using a predefined PICO‐based coding scheme. In cases of overlapping populations, the most comprehensive dataset was retained. Reviewers were blinded to author names, institutions, and journals during selection. Inter‐rater agreement was assessed with Cohen's *κ* (*κ* = 0.93 for titles/abstracts, *κ* = 0.97 for full texts).

### 2.5 Data Collection

Two reviewers (F.F. and M.C.) independently extracted data using a standardized form, piloted and refined by the review team. Disagreements were resolved by consensus. Corresponding authors were contacted twice (2‐week interval) to retrieve missing or unclear data; unresolved data were excluded.

### 2.6 Data Extraction

- Study characteristics: authors, year, design, setting, sample size, follow‐up, funding, and level of analysis.
- Population characteristics: age, sex, smoking status, periodontal status, implant characteristics (location, restoration type).
- Outcomes: incidence of peri‐implantitis and secondary outcomes (implant loss, mucositis, peri‐implant PPD, MBL changes).

For each study, reviewers recorded the level at which exposure and outcomes were assessed and reported (patient level, implant level). Reported effect estimates were extracted together with their measure type (odds ratio [OR], hazard ratio [HR], risk ratio), adjustment status, covariates included in the model, and the time‐point or follow‐up period to which they referred.

### 2.7 Risk of Bias Assessment

Two reviewers (A.C. and M.C.) independently evaluated risk of bias. Newcastle–Ottawa Scale (NOS) [27] was used for observational studies, with separate domain assessments for cohort and case–control designs. Overall methodological quality was categorized as high (7–9 points), moderate (4–6 points), or low (0–3 points). Disagreements were resolved through discussion with a third reviewer (G.B.). Inter‐reviewer reliability was excellent (*κ* = 0.98).

### 2.8 Data Synthesis

Given the limited number of eligible studies and the substantial methodological heterogeneity across investigations, the primary synthesis was conducted qualitatively. A quantitative synthesis was deemed methodologically inappropriate as the primary analytical strategy because of heterogeneity in the definitions of residual PPDs, diagnostic criteria for peri‐implantitis, follow‐up duration, level of analysis, and type of reported effect estimates.

For each study, the study design, follow‐up duration, periodontal exposure definition, peri‐implantitis case definition, level of analysis, available effect estimates, and direction of association between residual PPDs and peri‐implantitis were described. Particular attention was given to whether residual periodontal PPD were assessed at the patient or site level, and whether peri‐implantitis outcomes were reported at the patient or implant level.

Reported effect estimates, including ORs and HRs, were extracted and descriptively presented when available. These measures were not statistically pooled because they reflected different estimands, levels of analysis, and follow‐up structures. When raw 2 × 2 implant‐level data were available, crude odds ratios were calculated exclusively to facilitate descriptive comparisons across studies; these estimates were considered exploratory and were not used as the primary basis for inference.

The consistency of findings across studies was qualitatively evaluated according to the direction of association, magnitude of reported estimates, biological plausibility, and methodological limitations. No formal assessment of publication bias was performed because fewer than 10 studies were available and funnel plot‐based methods would therefore have been unreliable.

The interpretation and certainty of the available evidence were considered in light of risk of bias, heterogeneity in exposure and outcome definitions, residual confounding, variability in follow‐up duration, and the nonindependence of implants within the same patient when implant‐level analyses were reported. Failure to account for within‐patient clustering was considered a potential source of artificially inflated statistical precision and distorted effect estimates.

## 3 Results

### 3.1 Study Selection

**FIGURE 1:**
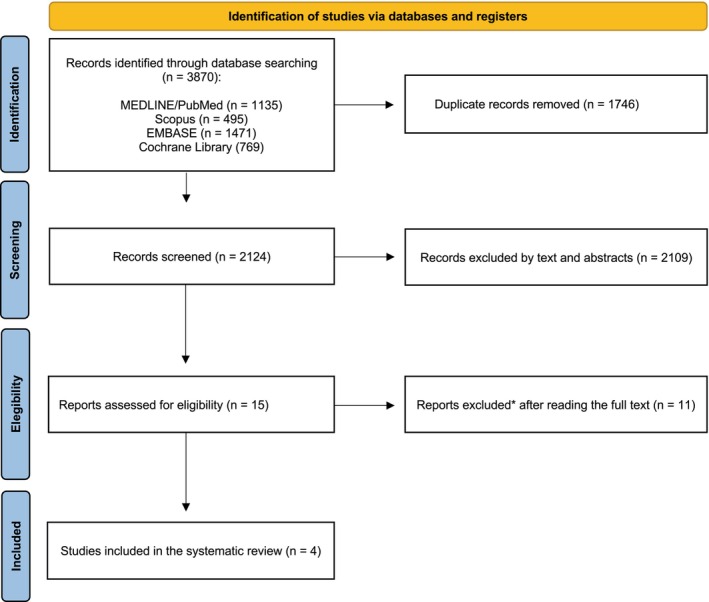
PRISMA flowchart illustrating the experimental study search and selection process. *The reasons for the exclusion are provided in the Supporting Information 2. *Source:* Page et al. [25].

The initial search generated a total of 3870 articles from all databases combined. Following duplicates removal (1746) and screening of titles and abstracts (2109), 15 articles qualified for full‐text screening (Figure 1). The reason for exclusion of the full‐texts is presented in Supporting Information 2. The Cohen's *κ* value for inter‐reviewer agreement was 0.93 at the first stage of screening and 0.97 at the second stage. Eleven articles were removed after full‐text assessment. Finally, four articles met all of the inclusion criteria and were included in the qualitative analysis [21, 28, 29, 30].

### 3.2 Characteristics of the Included Studies

The characteristics of the included studies are summarized in Table 1 and Supporting Information 3. All included studies were published between 2012 and 2021. Two investigations were conducted in Europe (France and Switzerland), one in Asia (China), and one in North America (United States). Overall, the studies included between 70 and 100 patients and between 165 and 260 implants. One study had a prospective design, whereas the remaining investigations were retrospective cohorts. Follow‐up duration ranged from 12 to 276 months. The included populations mainly consisted of partially edentulous adults with a history of treated moderate‐to‐severe periodontitis undergoing implant therapy and enrolled in SPC/SPIC programs. Mean patient age ranged from 44.5 to 68.9 years, and smokers were included in all cohorts. Implant placement protocols varied across studies and included both one‐stage [21, 29] and two‐stage surgical approaches [30].

**TABLE 1:** Methodological characteristics of the selected studies.

References	Year	Study design	Follow‐up (months)	Setting, country	Mean age (years)	Gender (% female)	Smokers (%)	No. of patients/implants	Periodontitis definition	Peri‐implantitis case definition	SPIC protocol
Kumar et al.	2018	Retrospective	> 60	University, USA	NA	52%	11.0%	86/222	NA	According to Lindhe and Meyle [31]	At least once per year
Pjetursson et al.	2012	Retrospective	Mean: 94.8 Range: 36–276	University, Switzerland	NA	NA	27.4%	70/165	According to Tonetti and Claffey [32]	Level 1: PPD ≥ 5 mm, BoP+ Level 2: PPD ≥ 6 mm, BoP+, MBL ≥ 2 mm	47 patients (67.1%) at the University, 23 (32.9%) in private practice setting; frequency not reported
Vagia et al.	2021	Retrospective	> 36	University, France	66.6 ± 8.2	52.33%	12.79%	86/260	According to Armitage [33]	PIKA: According to Karoussis et al. [34] PIBE: According to Berglundh et al. [1]	Every 3–6 months
Zhang et al.	2018	Prospective	Range: 12–60	Hospital, China	44.5 ± 9.4	50%	24.0%	100/214	According to Eke et al. [35]	According to Berglundh et al. [1]	Exclusion of patients who cannot keep SPIC; frequency of SPC not reported

Abbreviations: BoP, bleeding on probing; MBL, marginal bone loss; NA, nonavailable; PPD, probing pocket depth; SPIC, supportive peri‐implant care.

Several case definitions of periodontitis were used, none of which was the one provided by the 2017 World Workshop on the Classification of Periodontal and Peri‐Implant Diseases [36]. Residual PPDs represented the main exposure variable across studies, although definitions varied considerably, with PPD thresholds ranging from ≥ 3 to ≥ 6 mm. Some investigations further stratified residual pocket severity according to the number or percentage of sites affected. Additional periodontal variables included bleeding indices, clinical attachment loss, bone loss/age ratio, and periodontal diagnosis severity. No study specifically evaluated furcation involvement at adjacent teeth as a risk factor for peri‐implantitis.

Considerable heterogeneity was also observed regarding peri‐implantitis case definitions. Definitions generally relied on combinations of PPD, bleeding on probing and/or suppuration, together with radiographic bone loss thresholds, although important differences existed among studies. In the study by Vagia et al. [21], peri‐implantitis was defined according to both Berglundh et al. [1] (in the paper referred to as PIBE) and Karoussis et al. [34], that is, PPD ≥ 5 mm with BOP/suppuration and radiographic bone loss ≥ 2 mm (referred to as PIKA). According to Pjetursson et al. [29], peri‐implantitis was characterized by marginal bone loss of ≥ 2 mm beyond initial bone remodeling, BoP, and PPD ≥ 5 mm (level 1) or PPD ≥ 6 mm (Level 2). Kumar et al. [28] adopted the case definition proposed by Lindhe and Meyle [31] PPD > 4 mm, BOP/suppuration, and radiographic bone loss ≥ 2 mm. Zhang et al. [30] used a definition aligned with the 2017 World Workshop criteria, including bleeding and/or suppuration on probing, increased probing depth, and radiographic bone loss ≥ 3 mm apical to the most coronal portion of the implant [1]. Peri‐implantitis outcomes were assessed at either patient or implant level depending on the study design.

Several additional variables potentially influencing peri‐implantitis risk were considered across studies, including smoking status, implant‐related characteristics, and SPC/SPIC protocols. Regarding the latter, only one study explicitly reported the frequency of patient recalls, with SPC intervals ranging from 3 to 6 months according to periodontal status and treatment outcomes [21]. In the study by Kumar et al., all patients reported attending at least annual dental maintenance visits for SPC [28]. Pjetursson et al. reported that, following completion of treatment, 47 patients (67.1%) attended the SPC program at the university clinic, whereas 23 patients (32.9%) were referred back to private practitioners [29]. Zhang et al. excluded patients unable to adhere to periodontal and peri‐implant maintenance care, although SPC frequency was not specifically reported [30].

### 3.3 Main Findings of the Included Studies

The main findings and the outcomes of the included studies are reported in Table 2.

**TABLE 2:** Outcomes of the selected studies.

References	Implant survival rate	Peri‐implantitis occurrence (implant‐level)	Clustering accounted for	Risk estimates for residual PPD at baseline compared to controls (95% CI)	Main findings
Kumar et al.	NA	NA	Yes (mixed‐effects modeling)	OR: 2.73 (1.45, 5.16)	Residual PPD at implant placement were associated with peri‐implantitis occurrence. Inflammatory periodontal conditions at adjacent teeth further increased peri‐implantitis risk (OR = 8.0).
Pjetursson et al.	95.8%	Level 1: 22.2% Level 2: 8.8%	No	NA	Patients developing peri‐implantitis presented significantly more residual PPD after periodontal therapy than patients without peri‐implantitis (4.1 vs. 1.9 pockets). Implant‐level stratified data were not available.
Vagia et al.	99.2%	PIKA: 20.1% PIBE: 17.8%	Yes (patient‐level analyses)	PIKA HR: 1.06 (1.02–1.1) PIBE HR: 1.07 (1.03–1.12)	Residual PPD > 3 mm increased peri‐implantitis risk according to both peri‐implantitis definitions.
Zhang et al.	100%	11.2%	Yes (generalized estimating equation model)	HR: 3.62 (1.05, 12.51)	> 10% of sites with PPD ≥ 6 mm significantly associated with peri‐implantitis occurrence in multivariate model.

Abbreviations: NA, nonavailable; nPI, non‐peri‐implantitis; nRP, non‐residual pockets; PI, peri‐implantitis; PPD, probing pocket depth; RP, residual pockets.

Vagia et al. [21] retrospectively evaluated 86 patients rehabilitated with 260 implants and followed for a mean of 9.4 years, with 38.0% of patients presenting implants in function for at least 10 years. At implant placement, 50 patients (157 implants) presented residual PPD ≥ 5 mm, whereas 36 patients (103 implants) did not. At follow‐up, peri‐implantitis was diagnosed in 11 patients and 14 implants. Of these, 9 patients and 12 implants belonged to the residual pocket group, while only 2 patients and 2 implants without residual pockets developed peri‐implantitis. In Cox regression analyses, residual PPD before implant placement was significantly associated with peri‐implantitis occurrence. Specifically, the number of residual sites with PPD > 3 mm was associated with increased marginal peri‐implantitis risk according to both the PIKA (HR = 1.06; 95% CI: 1.02–1.10) and PIBE (HR = 1.07; 95% CI: 1.03–1.12) definitions.

Pjetursson et al. [29] retrospectively evaluated 70 treated periodontitis patients rehabilitated with 165 implants and followed for a mean of 7.9 years (range: 36–276 months). The cumulative implant survival rate was 95.8% (95% CI: 90.9%–98.1%). Patients who developed peri‐implantitis during follow‐up presented significantly more residual PPD ≥ 5 mm after APT compared with patients without peri‐implantitis (mean: 4.1 vs. 1.9 residual pockets). However, implant‐level stratified data according to residual pocket status at implant placement were not reported, precluding separate evaluation of peri‐implantitis occurrence in exposed and non‐exposed groups.

Kumar et al. [28] retrospectively evaluated 86 patients rehabilitated with 222 implants and followed for at least 60 months. At implant placement, 37 patients (108 implants) presented residual PPD ≥ 5 mm, whereas 49 patients (114 implants) did not. At follow‐up, peri‐implantitis was diagnosed in 129 implants, while 93 implants remained free of disease. Among implants affected by peri‐implantitis, 76 had been placed in patients with residual pockets and 53 in patients without residual pockets at baseline. The authors further reported that the presence of either gingivitis or periodontitis on adjacent teeth at the time of implant restoration was significantly associated with peri‐implantitis occurrence (OR = 8.0; 95% CI: 1.2–70.1).

Zhang et al. [30] prospectively evaluated 100 patients rehabilitated with 214 implants and followed for 12–60 months. Residual periodontal inflammation at implant placement was assessed according to mean PPD, proportion of sites with PPD ≥ 6 mm, and full‐mouth bleeding score. Among the 24 implants that developed peri‐implantitis during follow‐up, 21 had been placed in patients presenting > 10% of sites with PPD ≥ 6 mm, whereas only 3 belonged to patients with ≤ 10% of such sites. Conversely, among the 190 implants free of peri‐implantitis at follow‐up, 97 had been placed in patients with > 10% of sites with PPD ≥ 6 mm and 93 in patients with ≤ 10% of sites with deep residual pockets. Overall, the cumulative incidence of peri‐implantitis ranged from 11.2% at implant level to 16% at patient level. In multivariate analyses, the presence of > 10% residual sites with PPD ≥ 6 mm at implant placement was significantly associated with peri‐implantitis occurrence (HR = 6.71; 95% CI: 1.84–24.47).

### 3.4 Risk of Bias

All the retrospective studies were considered to be at moderate risk of bias [21, 28, 29], while the prospective study was judged as at low risk of bias [30] (Supporting Information 4).

## 4 Discussion

The present systematic review investigated whether, in patients with periodontitis, incomplete or suboptimal periodontal outcomes after APT before implant placement may influence the subsequent occurrence of peri‐implantitis in patients with treated periodontitis. Although based on a limited number of observational studies with heterogeneous methodologies, the available evidence overall suggested a relatively consistent association between less favorable periodontal treatment outcomes—particularly the persistence of residual deep PPDs—and increased peri‐implantitis occurrence during follow‐up.

A recurrent finding across the included studies was that both the extent and severity of residual PPDs appeared to influence peri‐implant outcomes. In particular, studies evaluating patients with a higher proportion of residual deep pockets after APT generally reported a greater occurrence of peri‐implantitis over time. Zhang et al. [30] observed that the presence of residual PPD ≥ 6 mm affecting > 10% of sites was associated with a substantially increased peri‐implantitis risk over a follow‐up of up to 5 years in patients with severe periodontitis. Similarly, Vagia et al. [21] reported in a long‐term retrospective cohort (mean follow‐up: 9.4 years) that the proportion of residual sites with PPD > 3 mm significantly influenced peri‐implantitis occurrence in adjusted Cox regression analyses. In addition, Pjetursson et al. found that patients who developed peri‐implantitis presented significantly more residual PPD ≥ 5 mm after completion of APT than patients maintaining peri‐implant health during follow‐up. Although direct comparisons among studies remain difficult because of methodological heterogeneity, the overall direction of the findings appeared relatively consistent.

Beyond the overall burden of residual periodontal inflammation, the review also identified signals suggesting a potential role of site‐specific periodontal inflammatory conditions adjacent to implant sites. Kumar et al. reported that inflammatory periodontal conditions affecting teeth adjacent to implants at the time of prosthetic delivery were associated with increased peri‐implantitis occurrence in partially dentate patients rehabilitated with non‐splinted implants. These observations may support the hypothesis that residual periodontal inflammation in close proximity to implant sites could contribute to the persistence or transmission of dysbiotic microbial and inflammatory conditions within the peri‐implant environment [22, 37, 38, 39]. However, given the retrospective nature of the available evidence and the limited number of studies, these findings should be interpreted cautiously and considered hypothesis‐generating rather than conclusive.

The interpretation of the available evidence should also consider several important methodological aspects. Definitions of residual PPD differed substantially across studies, with residual PPD thresholds ranging from 4 to 6 mm. Similarly, peri‐implantitis case definitions varied according to probing depth and radiographic bone loss thresholds. A negative relationship between bone loss thresholds and peri‐implantitis prevalence has previously been described [3, 19], such that more stringent diagnostic criteria generally yield lower peri‐implantitis rates. Furthermore, diagnostic approaches emphasizing radiographic bone loss may alter the distribution of mild and severe peri‐implantitis cases compared with definitions more strongly based on probing parameters. Consequently, the selected diagnostic thresholds may substantially influence the sensitivity for detecting associations between residual periodontal disease and peri‐implantitis. Interestingly, studies adopting more inclusive peri‐implantitis thresholds appeared to identify stronger associations with residual PPDs [21, 29], whereas stricter definitions may reduce statistical power and attenuate detectable associations. A quantitative synthesis of the included studies was therefore precluded by substantial heterogeneity in both clinical and statistical reporting. Beyond the variability in exposure and outcome definitions, the relationship between residual periodontal disease and peri‐implantitis was expressed using heterogeneous analytical approaches, including mean differences in residual PPD counts [29], OR [28], and HRs [21] from logistic and Cox regression models [30].

From a biological perspective, the findings of the present review appear plausible within the current understanding of peri‐implant microbiology and periodontal–peri‐implant interactions [8, 40, 41]. Once implants emerge into the oral cavity following prosthetic rehabilitation, the peri‐implant sulcus becomes colonized by a microbiome sharing similarities with the gingival sulcus around natural teeth [37, 42]. Residual PPDs may therefore act as persistent reservoirs of dysbiotic biofilm and unresolved inflammation, potentially facilitating microbial transmission toward peri‐implant niches [38, 39, 42]. This concept is coherent with the well‐established association between history of periodontitis and peri‐implantitis risk reported in several observational studies [20, 34, 43, 44, 45, 46]. At the same time, studies evaluating patients during supportive care have suggested that adequate periodontal control and maintenance adherence may partially mitigate this increased susceptibility [47, 48]. Therefore, the independent contribution of residual periodontal inflammation after APT, beyond the broader effect of maintenance quality and patient susceptibility, remains incompletely understood [16, 49, 50].

It has long been recognized that achieving the proposed endpoints of periodontal therapy may be challenging, particularly in patients with severe or complex periodontitis [16, 17, 50, 51]. In clinical practice, implant rehabilitation is frequently considered in patients presenting residual PPDs after completion of APT [16, 49, 50]. The findings of the present review suggest that more favorable periodontal treatment outcomes before implant placement may be associated with improved peri‐implant stability over time. However, the currently available evidence does not allow identification of definitive periodontal threshold values that could reliably predict peri‐implant outcomes at the individual patient level.

This review presents some limitations that should be acknowledged. From the perspective of the review process, the small number of available studies and the substantial heterogeneity in exposure definitions, peri‐implantitis case definitions, follow‐up duration, and supportive care protocols precluded quantitative synthesis and limited the possibility of drawing robust pooled estimates. In addition, no study specifically evaluated furcation involvement, despite its potential biological relevance. Important limitations also emerged from the primary studies themselves. Three of the four included investigations had a retrospective design [21, 28, 29], making them inherently more susceptible to selection bias and incomplete exposure ascertainment compared with prospective cohorts. Considerable variability was also observed in the SPC/SPIC regimens adopted across studies, ranging from strict university‐based maintenance recalls every 3 months to self‐reported annual private dental visits. This heterogeneity makes it difficult to disentangle the independent biological effect of residual PPD from the influence of maintenance adherence and quality of supportive care [47, 52, 53].

## 5 Conclusions

### 5.1 Implications for Clinical Practice

- The available observational evidence suggests a possible association between residual PPDs persisting after active periodontal therapy and peri‐implantitis occurrence in patients with treated periodontitis undergoing implant therapy.
- Although the current evidence base remains limited and heterogeneous, the findings are broadly consistent with existing recommendations emphasizing the importance of achieving periodontal stability before implant placement.
- Since complete elimination of residual PPDs may not always be clinically achievable, reinforcement of supportive periodontal and peri‐implant care protocols should be considered particularly in patients with severe or complex periodontitis.
- Careful periodontal re‐evaluation before implant rehabilitation may therefore be advisable, especially in patients presenting residual deep PPDs or other established risk determinants for peri‐implant diseases. The currently available evidence does not allow conclusions regarding the specific role of residual furcation involvement in peri‐implantitis risk.

### 5.2 Implications for Research

- Future studies should prioritize prospective designs with standardized definitions of both residual periodontitis and peri‐implantitis in order to improve comparability across investigations.
- The role of potential confounding variables, including smoking, systemic conditions, implant‐related characteristics, and adherence to SPC/SPIC, should be more comprehensively addressed in future multivariable models.
- SPC and SPIC regimens should be consistently reported, as maintenance‐related factors may influence peri‐implant outcomes and affect interpretation of the association with residual periodontal inflammation.
- Future investigations should evaluate not only residual PPD but also the potential role of furcation involvement and other site‐specific periodontal conditions in relation to peri‐implant disease development.
- Standardized and rigorous periodontal assessment before implant placement should be systematically incorporated into prospective studies and RCTs investigating implant therapy outcomes. In particular, detailed reporting of residual PPD and achievement of established endpoints of active periodontal therapy may help clarify their potential relationship with peri‐implant biological complications.
- Standardized reporting of periodontal parameters at implant placement may facilitate the development of more robust multivariable models and improve understanding of the relationship between residual periodontal disease and peri‐implant biological complications.

## Author Contributions

F.F., G.B., and M.A. made substantial contributions to conception of the study. F.F., G.B., A.C., and M.C. contributed to the study design. F.F., A.C., and M.C. searched and collected the data. G.B., A.C., S.C., and E.C. performed data analysis and interpretation. F.F., A.C., M.L.‐V., and M.C. prepared the first draft of the manuscript. All authors have read, revised critically, and approved the final manuscript.

## Funding

The authors have nothing to report.

## Ethics Statement

The authors have nothing to report.

## Consent

The authors have nothing to report.

## Conflicts of Interest

The authors declare no conflicts of interest.

## Supporting information


**Supporting Information: 1.** Search strategy developed for online databases.

**Supporting Information: 2.** Studies excluded after full text reading, with reason for exclusion.

**Supporting Information: 3.** Additional information related to study population and exposures.

**Supporting Information: 4.** Results of methodologic domains weighed by the Newcastle–Ottawa scale (Wells et al.).

## Data Availability

The data that support the findings of this study are listed in the main manuscript and Supporting Information [Link], [Link], [Link], [Link].
