# Supplementary material for: Residual Periodontal Pockets at Implant Placement as Risk Indicator for Peri‐Implantitis: A Systematic Review
Source: Clin Implant Dent Relat Res. 2026 Jul 27;28(4):e70174. doi: 10.1111/cid.70174 (PMC13408331; doi:10.1111/cid.70174)
Supplement: Supplementary file 1 — Supporting Information: 1. Search strategy developed for online databases. [file CID-28-0-s001.docx]

| (((((((“dental implants”[MeSH Terms] OR (“dental”[All Fields] AND “implants”[All Fields]) OR “dental implants”[All Fields]) OR ((“titanium”[MeSH Terms] OR “titanium”[All Fields]) AND (“dental implants”[MeSH Terms] OR (“dental”[All Fields] AND “implants”[All Fields]) OR “dental implants”[All Fields]))) AND (implant-abutment[All Fields] AND connection[All Fields])) OR (machined[All Fields] AND collar[All Fields])) OR microgap [All Fields]) OR (implant[All Fields] AND abutment[All Fields] AND connection[All Fields])) OR (crestal[All Fields] AND (“bone and bone- s”[MeSH Terms] OR (“bone”[All Fields] AND “bones”[All Fields]) OR “bone and bones”[All Fields] OR “bone”[All Fields]) AND level[All Fields])) AND (“1990/01/01”[PDAT]: “2017/05/27”[PDAT]) AND “humans”[MeSH Terms] | PUBMED |
| --- | --- |
| ‘dental’/exp OR dental AND (‘implants’/exp OR implants) OR ‘titanium’/exp OR tita- nium AND (‘dental’/exp OR dental) AND (‘implants’/exp OR implants) AND ‘implant abutment’ AND (‘connection’/exp OR connection) OR machined AND (‘collar’/exp OR collar) OR microgap OR ‘implant’/exp OR implant AND abutment AND (‘connection’/exp OR connection) OR crestal AND (‘bone’/exp OR bone) AND level AND [1-1-1990]/sd NOT [27-5-2017]/sd AND [1990–2017]/py. | EMBASE |
| (TITLE-ABS-KEY(peri-implantitis OR perimplantitis OR "peri-implant disease" OR "peri-implant diseases")) AND (TITLE-ABS-KEY("periodontal disease" OR periodontitis OR "periodontal status" OR "periodontal therapy")) AND (TITLE-ABS-KEY("clinical trial" OR "randomized clinical trial" OR "randomised clinical trial" OR "controlled clinical trial" OR "prospective studies" OR "cohort studies" OR "prospective cohort" OR "cross sectional studies" OR "case series" OR "retrospective studies" OR rcts OR ccts)) AND (LIMIT-TO(LANGUAGE, “English")) | SCOPUS |
| (peri-implantitis OR perimplantitis OR peri-implant disease OR peri-implant diseases) AND (periodontal disease OR periodontitis OR periodontal status OR periodontal therapy) | COCHRANE LIBRARY |
