# Supplementary material for: Residual Periodontal Pockets at Implant Placement as Risk Indicator for Peri‐Implantitis: A Systematic Review
Source: Clin Implant Dent Relat Res. 2026 Jul 27;28(4):e70174. doi: 10.1111/cid.70174 (PMC13408331; doi:10.1111/cid.70174)
Supplement: Supplementary file 2 — Supporting Information: 2. Studies excluded after full text reading, with reason for exclusion. [file CID-28-0-s003.docx]

Supplementary material 2 - Studies excluded after full text reading, with reason for exclusion.

| Reference | Reason For Exclusion |
| --- | --- |
| Gatti et al. (2008) | No PPD available |
| Guarnieri et al. (2021) | Implants placed in patients without RP |
| Karoussis et al. (2003) | Probably not RP at baseline |
| Lee et al. (2012) | PPD recorded at follow-up, not at implant placement |
| Meyle et al. (2014) | No data available regarding the aim of the study |
| Roccuzzo et al. (2022) | Missing periodontal data before implant placement |
| Rusu et al. (2023) | Implant placed before or after periodontal therapy |
| Sarbacher et al. (2022) | PPD recorded at follow-up, not at implant placement |
| Serrano et al. (2022) | Only 1-year follow-up |
| Swierkot et al. (2012) | Implant placed in patients without RP |
| Wahlström et al. (2010) | Missing periodontal data before implant placement |

PPD, Periodontal Pocket Depth; RP, Residual Pockets
