# Supplementary material for: Residual Periodontal Pockets at Implant Placement as Risk Indicator for Peri‐Implantitis: A Systematic Review
Source: Clin Implant Dent Relat Res. 2026 Jul 27;28(4):e70174. doi: 10.1111/cid.70174 (PMC13408331; doi:10.1111/cid.70174)
Supplement: Supplementary file 3 — Supporting Information: 3. Additional information related to study population and exposures. [file CID-28-0-s004.docx]

**Additional information related to study population and exposures**

| **Reference** | **Implant placement procedure (stage, augmentation procedures, bone/tissue level)** | **Implant location site** | **N° of residual pockets** | **Residual pockets threshold** |
| --- | --- | --- | --- | --- |
| Kumar et al. | NA | NA | NA | NA |
| Pjetursson et al. | Tissue level with a 2.8 mm machined neck | Anterior: 21.7% Premolar: 46.0% Molar: 32.3% | Peri-implantitis Level 1: 4.1 (3.1%)  Peri-implantitis Level 2: 3.4 (2.5%)  Non-peri-implantitis: 1.9 (1.5%) | PPD ≥ 5 mm |
| Vagia et al. | Implants placed at the end of APT or during SPT | NA | PPD > 3 mm  11.30 NOPIKA  24.68 PIKA  11.21 NOPIBE  28.07 PIBE  PPD > 4 mm  5.23 NOPIKA  10.08 PIKA  5.35 NOPIBE  10.25 PIBE | PPD > 3 mm  PPD > 4 mm |
| Zhang et al. | Two-stage protocol, sandblasted surface, GBR or sinus lift when indicated, bone level (platform 2-3 mm subcrestal) | Maxilla: 49.07%, Mandibula: 50.93%; Anterior: 51.40%  Posterior: 48.60% | PPD ≥ 6 mm (%), number and % of implants  Cases: 3 (12.50%) ≤ 10%, 21 (87.50%) > 10% Controls: 93 (48.85%) ≤ 10%, 97 (51.05%) > 10% Overall: 96 (44.86%) ≤ 10%, 118 (55.14%) > 10% | PPD ≥ 6 mm |

| **Reference** | **N° of implants in RP (PPD ≥ 5 mm) group** | **N° of implants in nRP (PPD ≥ 5 mm) group** | **N° of implants in PI group** | **N° of implants in nPI group** | **N° of implants in RP-PI group** | **N° of implants in nRP-PI group** | **N° of implants in RP-nPI group** | **N° of implants in nRP-nPI group** |
| --- | --- | --- | --- | --- | --- | --- | --- | --- |
| Kumar et al. | 108 (48.6%) | 114 (51.4%) | 129 (58.1%) | 93 (41.9%) | 76 (34.2%) | 53 (23.9%) | 32 (14.4%) | 61 (27.5%) |
| Pjetursson et al. | NA | NA | NA | NA | NA | NA | NA | NA |
| Vagia et al. | 157 (60.4%) | 103 (39.6%) | 14 (5.4%) | 246 (94.6%) | 12 (4.6%) | 2 (0.8%) | 145 (55.8%) | 101 (38.8%) |
| Zhang et al. | 118 (55.1%) | 96 (44.9%) | 24 (11.2%) | 190 (88.8%) | 21 (9.8%) | 3 (1.4%) | 97 (45.3%) | 93 (43.5%) |

**Outcome raw data both derived from original studies and provided by the authors**

Abbreviations: RP, Residual Pockets; nRP, non-Residual Pockets; PI, Peri-Implantitis; nPI, non-Peri-Implantitis; PPD, Probing Pocket Depth; NA, non-available.
