# Supplementary material for: Residual Periodontal Pockets at Implant Placement as Risk Indicator for Peri‐Implantitis: A Systematic Review
Source: Clin Implant Dent Relat Res. 2026 Jul 27;28(4):e70174. doi: 10.1111/cid.70174 (PMC13408331; doi:10.1111/cid.70174)
Supplement: Supplementary file 4 — Supporting Information: 4. Results of methodologic domains weighed by the Newcastle–Ottawa scale (Wells et al.). [file CID-28-0-s002.docx]

|  | Selection | | | | Comparability | | Exposure | | | **TOTAL** |
| --- | --- | --- | --- | --- | --- | --- | --- | --- | --- | --- |
|  | 1 | 2 | 3 | 4 | 1a | 1b | 1 | 2 | 3 |  |
| Pjetursson et al., 2012 | A | B | B | A |  |  | A | A | B | **4** |
|  | 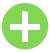 | 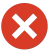 | 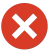 | 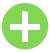 | 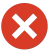 | 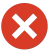 | 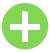 | 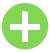 | 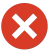 | **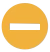** |
| Kumar et al., 2018 | A | B | B | A |  |  | A | A | B | **6** |
|  | 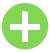 | 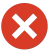 | 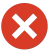 | 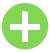 | 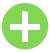 | 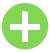 | 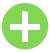 | 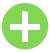 | 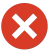 | **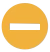** |
| Vagia et al., 2021 | A | B | B | A |  |  | A | A | B | **6** |
|  | 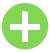 | 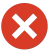 | 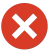 | 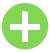 | 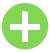 | 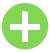 | 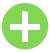 | 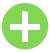 | 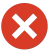 | **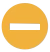** |

- Selection

1. Is the case definition adequate?
2. Representativeness of the cases
3. Selection of controls
4. Definition of controls

- Comparability

1. Comparability of cases and controls on the basis of the desing or analysis

- Exposure

1. Ascertainment of exposure
2. Same method of ascertainment for cases and controls
3. Non-response rate

|  | Selection | | | | Comparability | | Exposure | | | **TOTAL** |
| --- | --- | --- | --- | --- | --- | --- | --- | --- | --- | --- |
|  | 1 | 2 | 3 | 4 | 1a | 1b | 1 | 2 | 3 |  |
| Zhang et al., 2018 | B | A | A | A |  |  | B | A | D | **8** |
|  | 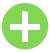 | 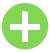 | 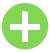 | 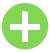 | 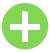 | 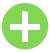 | 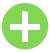 | 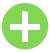 | 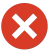 | **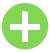** |
| Judgement | |  |  |  |  |  |  |  |  |  |
| 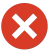 | 0-3 High |  |  |  |  |  |  |  |  |  |
| 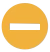 | 4-6 Moderate |  |  |  |  |  |  |  |  |  |
| 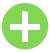 | 7-9 Low |  |  |  |  |  |  |  |  |  |

- Selection

1. Representativeness of the exposure cohort
2. Selection of the non exposure cohort
3. Ascertainment of exposure
4. Demonstration that outcome of interest was not present at start of study

- Comparability

1. Comparability of cohorts on the basis of the design or analysis

- Outcome

1. Assessment of outcome
2. Was follow-up long enough for outcome of interest
3. Adequacy of follow-up cohort
